# Bioactive Compounds and Antioxidant, Antiperoxidative, and Antihemolytic Properties Investigation of Three *Apiaceae* Species Grown in the Southeast of Morocco

**DOI:** 10.1155/2020/3971041

**Published:** 2020-09-22

**Authors:** Mgal Derouich, Eimad Dine Tariq Bouhlali, Mohamed Bammou, Abdelbasset Hmidani, Khalid Sellam, Chakib Alem

**Affiliations:** ^1^Biochemistry of Natural Products, Faculty of Sciences and Techniques, Moulay Ismail University, 52000 Errachidia, Morocco; ^2^Biology, Environment and Health Team, Faculty of Sciences and Techniques, Moulay Ismail University, 52000 Errachidia, Morocco; ^3^National Institute for Agricultural Research, Regional Center of Errachidia, 52000 Errachidia, Morocco

## Abstract

For a long time, *Apiaceae* species have been widely employed in the southeast of Morocco for culinary and folk healing purposes. In the current study, we investigated three *Apiaceae* herbs known as coriander (*Coriandrum sativum*), celery (*Apium graveolens*), and parsley (*Petroselinum crispum*) for their antioxidant, antiperoxidative, and antihemolytic properties. The HPLC-DAD has been used to classify and measure phenolic compounds. The major phenolic compounds studied were p-coumaric, chlorogenic, caffeic acids, luteolin, and quercetin. The polyphenol level was also estimated via Folin–Ciocalteu's method, aluminium chloride, and acidified vanillin. Parsley showed the highest polyphenol level and, thus, showed potential antioxidant activities demonstrated by DPPH, ABTS scavenging tests, and reducing power (FRAP), as well as TBARS assays. Very strong correlations were depicted among phenol levels and antioxidant assays (*R*^2^ ≥ 0.910) and among antihemolytic activity and flavonoids (*R*^2^ ≥ 0.927), indicating the implication of phenolic compounds, mainly flavonoids, in the antiradical properties. These finding may prove the traditional use of these *Apiaceae* species in the management of numerous disorders cited within the Moroccan pharmacopoeia.

## 1. Introduction

During pathological regulation, the mitochondrial proteins involved in electron transfer can overproduce oxygen free radicals, which are prone to alter DNA, peptides, and lipids, leading to cell ageing, cardiovascular diseases, inflammation-related diseases, and cancer [1]. However, equilibrium between antioxidants and oxidants is recognized to be efficient in ensuring a healthy biological system [2]. Indeed, when the immune system interacts with xenobiotic agents, high levels of oxygen prooxidants (ROS) are produced and are not properly neutralized by antioxidant enzymes such as glutathione peroxidase and catalase, which lead to their accumulation in the mitochondria, thereby compromising their physiological role [3, 4]. There is, therefore, a state of oxidative stress that stimulates the development, progression, and complication of metabolic syndrome, neurodegenerative and inflammatory diseases, and cancer [5–7]. Nowadays, most studies have shown a strong negative association between various chronic pathologies and a high-fruit and vegetable diet, which retains fiber, essential nutrients, and beneficial bioactive compounds [8, 9]. Free-radical scavengers from food and diets can strengthen cell antioxidant powers and protect its interior and exterior components from oxidation-related alterations [10]. Moreover, free-radical trappers have been intensely employed to prolong the shelf-life of various dietary substances. Although, there is a common view that many artificial antioxidants such as butylhydroxyanisole and butylhydroxytoluene (BHA and BHT, respectively) used to process food must be switched by natural antioxidants since they were entitled to show potential toxicity and health hazards [11].

As antioxidants, polyphenols from plants can rule out the deleterious effects of oxidation through many ways including chelating metal ions, preventing the accumulation of ROS, empowering antioxidant enzymes, and inhibiting lipid peroxidation [12]. Within various plants, some original and comestible species are of tremendous importance, given the fact that they can be employed to make up raw materials and formulations rich in nutrients with health benefits. Members of the carrot family (Apiaceae) synthesize large series of phytonutrients in which polyphenols were reported to have multiple advantageous effects comprising antioxidant activity [13]. In fact, celery (*Apium graveolens*), coriander (*Coriandrum sativum*), and parsley (*Petroselinum crispum*), species among the *Apiaceae* family, are aromatic plants of the Mediterranean flora and are notoriously employed as culinary herbs and folk therapeutic remedies. In the Moroccan traditional pharmacopoeia, many genera of this family were used mainly to treat various ailments and symptoms, including chills, dyspepsia, cough, bites of poisonous animals, intestinal pain, mumps, infertility, allergies, epigastric abdominal pain, kidney symptom, ear pain, antepartum bleeding, and reduced sexual desire [14].

To date, there is no appropriate method to evaluate the antiradical ability within various herb extracts; thus, several tests were performed, comprising organic substrate-based assays such as 2,2-diphenyl-1-picrylhydrazyl (DPPH) and 2,2′-azinobis (3-ethylbenzothiazoline 6-sulfonate) (ABTS), mineral substrate-based tests such as ferric reducing antioxidant power (FRAP), and also biological substrate-based ones such as thiobarbituric acid reactive substances (TBARS) and 2,2,-azobis(2-amidinopropane) dihydrochloride- (AAPH-) induced hemolysis assays. The goal of the current investigation is to quantify the total phenolic, flavonoids, and tannin contents plus the antioxidant, antiperoxidative, and antihemolytic powers, employing DPPH, ABTS, FRAP, TBARS, and AAPH-induced hemolysis assays of three *Apiaceae* species used in food flavoring applications and as traditional healing remedies.

## 2. Materials and Methods

### 2.1. Chemicals and Reagents

Aluminium chloride (AlCl_3_), 4-hydroxy-3-methoxybenzaldehyde (Vanillin; V), quercetin-3-O-rutinoside (Rutin; RU), 3,4,5-trihydroxybenzoic acid (Gallic acid; GA), 2-(3,4-Dihydroxyphenyl)-3,4-dihydro-2H-chromene-3,5,7-triol (Catechin; C), sodium hydroxide (NaOH), sodium nitrite (NaNO_2_), 6-Hydroxy-2,5,7,8-tetramethylchromane-2-carboxylic acid (Trolox, T), hydrochloric acid (HCl), sodium carbonate (Na_2_CO_3_), 2,2-Diphenyl-1-picrylhydrazyl (DPPH), acetic acid (AcOH), trichloroacetic acid (TCA), 2,2′-azobis (2-methylpropionamidine) dihydrochloride (AAPH), potassium persulphate (K_2_S_2_O_8_), Sodium doDecyl Sulphate (SDS); sodium acetate buffer (NaOAc), 2,4,6-tri(2-pyridyl)-striazine (TPTZ), phosphate-buffered saline (PBS), L-Ascorbic acid (AA), Folin–Ciocalteu's reagent (FCR), and 2-thiobarbituric acid (TBA). The overall reagents mentioned were procured from Sigma-Aldrich (France). 2,2′-Azinobis(3-ethylbenzothiazoline-6-sulfonic acid) diammonium salt (ABTS), 1-butanol (n-BuOH), Iron (III) sulphate (FeSO_4_), methanol (MeOH), and ferric chloride (FeCl_3_) were purchased from VWR Prolabo (France).

### 2.2. Plant Materials

The *Apiaceae* plants celery (*Apium graveolens*), coriander (*Coriandrum sativum*), and parsley (*Petroselinum crispum*) were collected during the flowering period from the Tinghir region located in the southeast of Morocco (Latitude: 31° 30′ 53″ N, Longitude: −5° 31′ 58″ W, Altitude: 1300 m). Voucher specimens were used to identify plants and were deposited in the herb facility of the Faculty of Sciences and Techniques at Errachidia, Morocco. Plant samples were first dried and, then, stored away from light at room temperature (25°C) prior extraction.

### 2.3. Preparation of Rich Polyphenol Extracts

Coriander-, parsley-, and celery-rich polyphenol extracts preparation was performed following the procedure established by Bouhlali et al. [15] with slight adjustments. In summary, thirty grams of powdered aerial parts of plants was extracted using 150 mL MeOH–H_2_O (4 : 1, v/v), at 35°C for 12 h using an orbital shaker-incubator. After then, a liquid-liquid extraction using hexane was performed in order to remove hydrophobic pigments such as chlorophyll which has the same color as the extracts. Then, the solution was filtered, and the filtrate was concentrated using a rotary evaporator under minimal pressure at 40°C until total evaporation of the solvent. The resulting crude extracts were stored at −20°C in dark glass bottles until further use. The extracts were redissolved in a known dilution of water to determine their phenolic, flavonoidic, and tannin contents plus their antioxidant, antiperoxidative, and antihemolytic abilities.

### 2.4. Identification of Phenolic Acids and Flavonoids

The plant extracts were evaluated for their phenolic and flavonoid composition by means of high-performance liquid chromatography (HPLC) as described by Bouhlali et al. [16], with slight changes. One gram of the plant extract was first dissolved in 25 mL acidified methanol solution (1 N HCl/methanol/water, 1/80/19, v/v/v) and, then, ultrasonicated for 30 min. The mixture was centrifuged at 1000 g for 15 min, and 2 mL of the supernatant was filtered via a 0.45 *μ*m filter before the injection. Phenolic standards (including seven phenolic acids: p-coumaric, vanillic, ferulic, caffeic, syringic, chlorogenic, and gallic acids and three flavonoids: quercetin, luteolin, and rutin) were prepared at a stock solution of 100 *μ*g/mL and used to prepare standard plots. A Shimadzu liquid chromatography system (Kyoto, Japan) equipped with an auto-sampler (SIL-20A), dual pump (LC-20AB), a vacuum degasser (DGU-20A), diode array detector (SPD-M10A), and a system controller (SCL-10A) was implemented to conduct the analytical separation. Polyphenol compounds were separated at 40°C using the Restek C18 column (150 × 4.6 mm, 5 *μ*m particle size) (Bellefonte, Pennsylvania, USA). The mobile phase comprised water-acetic acid (97 : 3, v/v) (eluent A) and acetonitrile (eluent B). Elution was executed at a flow rate of 1 mL min−1 with the following gradient outline: 0–5 min, 0–8% solution B (linear gradient); 5–25 min, 8–25% solution B (linear gradient); 25–30 min, 25% solution B (isocratic elution); and 30–50 min, 25–90% solution B (linear gradient). The injection volume was 20 *μ*L, and the wavelengths of detection were set at 280, 320, and 350 nm. The compounds were authenticated with the help of retention time and comparing peak areas with those obtained under the same conditions. The quantification of the different samples was performed by the measurement of the integrated peak area, and the contents were calculated by means of the standard curve by plotting the peak area against concentration of the respective standard sample. Triplicate measurements were performed, and the results were averaged and reported as milligrams per 100 g DW of plant extracts.

### 2.5. Measurement of Phenol Components

Quantification of phenols within the three extracts was executed using Folin–Ciocalteu's reagent, following the protocol reported by Bouhlali et al. [17] whose principle relies on the decay of phosphomolybdic-phosphor tungstic acid caused by phenol compounds in an alkaline medium [18]. To proceed, 500 *µ*L of Folin–Ciocalteu's reagent diluted ten-fold in water was poured onto 100 *µ*L of a suitable diluted hydromethanolic plant extract and, then, 400 *µ*L of sodium carbonate solution (7.5% w/v). The resulting solution was allowed to stand for 60 min at room temperature, and its turbidity was noted at *λ* = 765 nm. The results were presented as gram of Gallic acid equivalent per 100 gram dry weight (g GAE.100 g^−1^ DW) through the calibration plot with gallic acid.

### 2.6. Measurement of the Flavonoid Content

The method of Kim et al. [19] was implemented to estimate the flavonoid amount. To start, four milliliter of distilled water was stirred with 1 mL of plant extract. Then, 0.3 mL sodium nitrite solution (5%) was added, followed by 0.3 mL of aluminium chloride solution (10%). Test tubes were incubated for 5 min at ambient temperature, and after that, 2 mL of sodium hydroxide (1 M) was stirred within the mixture. Test tubes were filled up to 10 mL using distilled water and, then, vortexed thoroughly, and the turbidity was read along with the blank at *λ* = 510 nm. Total flavonoid contents were presented as *g* of rutin equivalent per 100 grams dry weight (g RE.100 g^−1^ DW) through the calibration plot with Rutin.

### 2.7. Measurement of Total Condensed Tannins

Quantification of total condensed tannins was performed employing the method modified by Heimler et al. [20]. In brief, four hundred microliters of the plant extract was stirred with 3 mL of vanillin mixed with methanol (4%) and concentrated hydrochloric acid (1.5 mL). The mixture was incubated for 15 min at room temperature so that the reaction starts, and the turbidity was determined at *λ* = 500 nm. Total condensed tannin contents were depicted as *g* catechin equivalent per 100 gram dry weight (g CE.100 g^−1^ DW) through the calibration plot with Catechin.

### 2.8. Antioxidant Activities

#### 2.8.1. DPPH Radical Trapping Power

The electron-donating capacity of the obtained extracts was quantified by bleaching of the purple-colored solution of 1,1-diphenyl-2-picrylhydrazyl (DPPH^●^) radical following the procedure reported by Blois [21] with slight changes. The closely stable radical cation DPPH^●^ changes color from red to yellow when scavenged. Thus, it is used to quantify the antiradical activity, usually expressed as IC_50_ values (correspond to the antioxidant level needed to lower the initial concentration of DPPH^●^ by 50%). The lower IC_50_ value indicates a potent antioxidant effect [22]. To proceed, the reactants included 100 *µ*L of the plant extract at various concentrations and 1.9 mL of DPPH mixed with methanol (0.3 mM). The solution was strongly shaken and incubated at room temperature for twenty minutes. Later on, the turbidity was measured at *λ* = 517 nm. Vitamin C played the reference antioxidant. The antiradical activity was presented as IC_50_ (*µ*g/mL) which is the concentration required to cause 50% DPPH inhibition. The free-radical trapping capacity was, thus, calculated manipulating the following equation:

(1) $$\begin{array}{c} \left(\text{\%}\right)\text{Inhibition}=\frac{\left(\text{Abs}.\text{ contr}-\text{Abs}.\text{ extr}\right)}{\text{Abs}.\text{ contr}}\;\times 100, \end{array}$$

where Abs. contr is the turbidity of the control and Abs. extr is the turbidity of the plant extract.

#### 2.8.2. Iron Reducing Power (FRAP)

The potassium ferricyanide-ferric chloride method [23] was used to quantify reducing power whose principle relies on the reduction of Fe^3+^-TPTZ complex to its colored ferrous form (Fe^2+^-TPTZ) in the existence of antioxidant compounds. Hence, it serves as a significant indicator of antiradical potential. The FRAP reagent contained 50 mL of acetate buffer (0.3 M) at pH (3.6), 5 mL of TPTZ solution (10 mM) prepared in 5 mL of ferric chloride solution (FeCl_3_) (20 mM), and HCl (40 mM). Then, 2 mL of the FRAP solution was immediately poured on 10 µL of each extract. Test tubes were incubated for ten minutes at room temperature. Later on, the turbidity of the resulting mix was read along with the blank at *λ* = 593 nm. Trolox was implemented to prepare the calibration plot. The results of the three plant extracts were displayed as trolox equivalent in *µ*mol.g^−1^ dry weight (DW).

#### 2.8.3. ABTS Radical Trapping Assay

The ABTS scavenging capacity was assessed employing the ABTS^+●^ free-radical fading assay [24]. To proceed, we induced the production of ABTS^+●^ radical cations by the mixing aqueous solution of ABTS (7 mM) and potassium persulphate solution (2.45 mM). The upcoming mixture was incubated in dark at room temperature for twelve to sixteen hours prior to use. Distilled water was employed to dilute the solution in the objective of recording a turbidity of 0.700 ± 0.005 at 734 nm. Later on, 30 *µ*L of each plant extract was stirred up with 3 mL of ABTS^+●^ radical solution and, then, was left for 6 minutes at ambient temperature. Consequently, the turbidity at 734 nm was measured. A standard plot was constructed employing Trolox. The total antioxidants were depicted as *µ*mol trolox equivalents per gram dry weight plant material.

#### 2.8.4. Thiobarbituric Acid Reactive Substances (TBARS) Assay

The TBARS test was implemented to evaluate the antiperoxidative effect as described by Bouhlali et al. [25]. 500 *µ*L of egg yolk homogenate (10% w/v dissolved in phosphate-buffered saline (pH 7.4)) was stirred with 100 *µ*L of the plant sample, and then, the resulting solution was filled with distilled water up to 1000 *µ*L. Then, 50 *µ*L of iron (III) sulphate (FeSO_4_) (0.07 M) was poured on the solution which was consequently incubated for a half an hour at 37°C to start lipid peroxidation. To the overall solution, three components (50 *µ*L of 20% trichloroacetic acid, 1.5 mL of thiobarbituric acid (TBA) (0.8% w/v prepared in 1.1% sodium dodecyl sulphate) and 1.5 mL of acetic acid (20%, pH 3.5)) were added, mixed, and heated at 95°C during exactly one hour. Thereafter, following cooling at ambient temperature, 6 mL of 1-butanol was added to each test tube. The contents of the tubes were agitated, and to remove the precipitated protein, a centrifugation was performed at 1000 g for ten minutes; later on, the turbidity of the Malondialdehyde (MDA)-TBA complex in the resulting supernatant was read against 3 mL of 1-butanol at *λ* = 532 nm. Only distilled water (100 *µ*L) served as the negative control. Vitamin C was the standard. The antiperoxidative effect was deduced using the following equation:

(2) $$\begin{array}{c} \text{Inhibition of lipid peroxidation }\left(\text{ILP},\text{ \%}\right)=\frac{\left(\text{Abs}.\text{ control}-\text{Abs}.\text{ sample}\right)}{\text{Abs}.\text{ control}}\times 100. \end{array}$$

The ILP (%) plotted against the various concentrations of samples or standards and IC_50_ levels (concentration of sample or reference necessary to prevent 50% of lipid oxidation) were determined.

#### 2.8.5. AAPH-Induced Hemolysis Assay

The antiradical property of plant extracts was also assessed via the neutralization of a peroxyl radical initiator, AAPH, following the protocol established by Blache and Prost [26] with minor modifications. Briefly, 200 *µ*L of rabbit blood collected in heparinized bulbs was stirred up with 10 *µ*L of plant extract, and then, 600 *µ*L of AAPH (10%) was added. The resulting solution was slowly agitated and incubated at 37°C. Later on, after every 5 min, the turbidity of the solution was noted at *λ* = 540 nm. The water-methanol extract was replaced by saline (0.9% NaCl) and trolox in the negative and positive controls, respectively. The preventive powers of plant extracts against AAPH-induced erythrocyte oxidative hemolysis were determined via half-time hemolysis (HT_50_) which is the necessary time to prevent the hemolysis of 50% erythrocytes. Trolox played the standard.

### 2.9. Statistical Analysis

All measurements were performed six-fold. Data obtained were processed using SPSS 23 software. The observed values were depicted as the average of six repetitions for all assays ± SE (standard error). An ANalysis Of VAriance (ANOVA) was performed to investigate the difference among the experimental groups. A Kolmogorov–Smirnov test was performed to evaluate the normality of distribution. To analyze the pairwise variations among variables, post hoc Bonferroni's test was executed at the level of significance *α* = 0.001. Pearson's square correlation coefficient (*R*^2^) was implemented to evaluate the relation among two variables.

## 3. Results

### 3.1. Identification of Phenolic Acids and Flavonoids

**Figure 1:**
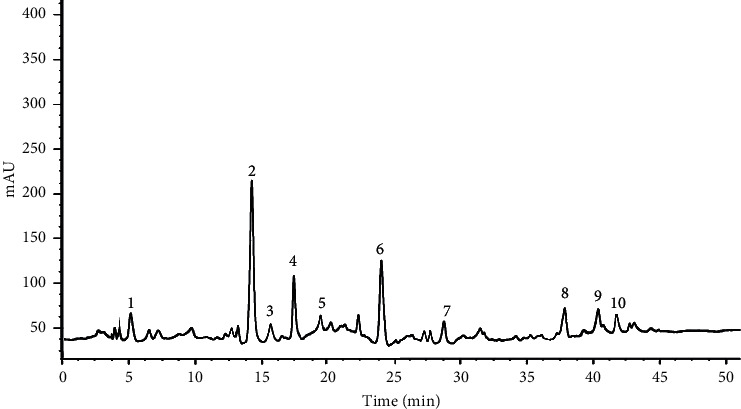
HPLC-DAD chromatograms of the parsley extract. Peak numbers: gallic acid (1); chlorogenic acid (2); vanillic acid (3); caﬀeic acid (4); syringic acid (5); p-coumaric acid (6); ferulic acid (7); rutin (8); luteolin (9); and quercetin (10).

The typical phenolic profiling of the studied plants extracts is revealed in Figure 1, and the results are illustrated in Table 1. The plant species exhibited significant differences (*p* < 0.001) concerning their content in phenolic and flavonoid compounds. All plant extracts were demonstrated to retain all the analyzed polyphenols and flavonoids at various concentrations. In the celery extract, p-coumaric acid was the predominant compound (114.30 ± 10.87 mg/100 g DW), tailed by chlorogenic acid (102.33 ± 10.02 mg/100 g DW), caffeic acid (67.65 ± 7.04 mg/100 g DW), luteolin (31.63 ± 3.51 mg/100 g DW), quercetin (25.08 ± 3.12 mg/100 gDW), gallic acid (24.36 ± 3.09 mg/100 g DW), ferulic acid (21.67 ± 3.56 mg/100g DW), rutin (16.73 ± 2.05 mg/100g DW), syringic acid (6.62 ± 0.93 mg/100 g DW), and vanilic acid (4.50 ± 0.99 mg/100 g DW). On the other hand, the parsley extract was characterized by the presence of cholorogenic acid as the predominant compound (171.30 ± 10.55 mg/100 g DW) and syringic acid as the minor compound (9.49 ± 0.77 mg/100 g DW). In the coriander extract, p-coumaric acid was the most abundant compound (91.18 ± 3.39 mg/100 g DW), followed by chlorogenic acid (82.30 ± 7.51 mg/100 g DW), caffeic acid (41.09 ± 5.14 mg/100 g DW), gallic acid (29.16 ± 3.23 mg/100 g DW), luteolin (27.98 ± 2 mg/100 g DW), vanilic acid (22.11 ± 2.59 mg/100 g DW), rutin (19.67 ± 2.89 mg/100 g DW), and quercetin (6.66 ± 0.30 mg/100 g DW).

Means in the identical line followed by different superscripts (a–d) are significantly different according to the post hoc Bonferroni test (*p* < 0.001).

### 3.2. Phenol Content, Flavonoids, and Total Condensed Tannin Contents

Results from the quantitative determination of total polyphenols, total condensed tannins, and total flavonoids of the three *Apiaceae* species are revealed in Table 2. The phenol amount varied largely (*p* < 0.001). The phenolic content increased in the order of coriander (1.372 g GAE/100 g) < celery (1.739 g GAE/100 g) < parsley (2.163 g GAE/100 g). The flavonoid content in all analyzed plant extracts varied considerably (*p* < 0.001) and ranged between 0.814–1.573 g RE/100 g. Parsley exhibited the richest level of flavonoid content and celery recorded the lowest level. Concerning condensed tannins, they showed significant (*p* < 0.001) differences among plant species. Celery showed the highest level (0.365 g CE/100 g) while the lowest level was observed in coriander (0.123 g CE/100 g).

### 3.3. Antioxidant Ability

#### 3.3.1. DPPH, ABTS, and FRAP Assays

**Figure 2:**
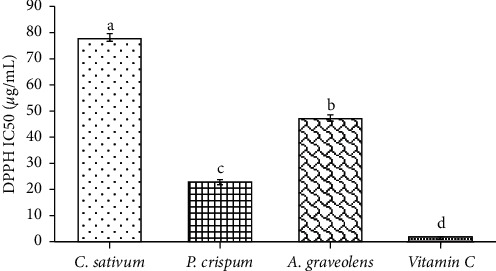
Antioxidant activity of the studied plant species based on DPPH assay. Amounts are mean (*n* = 6) ± SE. bars followed by different lower-case letters (a–d) which are significantly different (*p* < 0.001).

**Figure 3:**
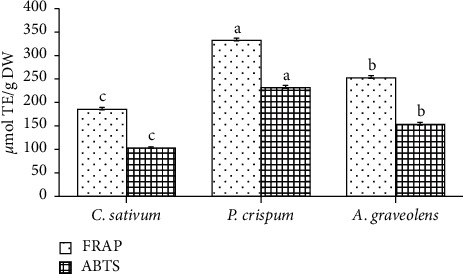
Antioxidant activity of the studied plant species based on FRAP and ABTS assay. Amounts are mean (*n* = 6) ± SE. bars followed by different lower-case letters (a–d) which are significantly different (*p* < 0.001).

Free-radicals trapping capacity was estimated using ABTS, DPPH, and reducing power assessed via FRAP assay. All analyzed *Apiaceae* species exhibited largely (*p* < 0.001) different scavenging potential. As revealed in Figures 2 and 3, parsley showed the strongest scavenging ability based on DPPH^IC50^ (22.84 *µ*g/mL) and ABTS (231.46 *µ*mol TE/g). Coriander recorded the weakest scavenging potential based on ABTS (102.95 *µ*mol TE/g) and DPPH^IC50^ (77.62 *µ*g/mL). Nevertheless, the latter remains lower compared to ascorbic acid (IC_50_ = 1.87 *µ*g/mL) being the reference antioxidant. In this study, DPPH^●^ and ABTS^●+^ radicals scavenging activities of the tested samples increased in the order: parsley > celery > coriander.

Concerning reducing power, the FRAP assay showed that parsley displayed the highest reducing activity (331.76 *µ*mol TE/g) followed by celery (252.05 *µ*mol TE/g) and, then, coriander (185.01 *µ*mol TE/g).

#### 3.3.2. Antiperoxidative Assay (TBARS)

**Figure 4:**
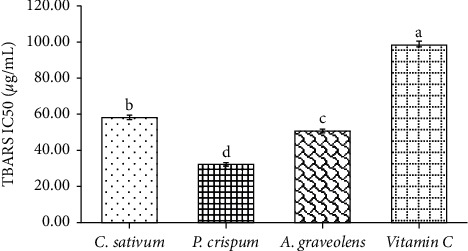
Antiperoxidative activity of different plant extracts. Amounts are mean (*n* = 6) ± SE. bars followed by different lower-case letters (a–d) which are significantly different (*p* < 0.001).

The results of this test reported in Figure 4 indicated that coriander, parsley, and celery extracts were demonstrated considerable (*p* < 0.001) discrepancies regarding the reduction of MDA formed by the oxidative action of FeSO_4_. Parsley exhibited the highest antioxidant power (IC_50_ = 32.19 *µ*g/mL) followed by celery and, then, coriander (IC_50_ = 58.04 *µ*g/mL). These findings are largely higher compared to ascorbic acid (IC_50_ = 98.25 *µ*g/mL) being the reference antioxidant.

#### 3.3.3. AAPH-Induced Hemolysis Assay

In the current study, coriander, celery, and parsley hydromethanolic extracts evidenced a large (*p* < 0.001) protective effect against AAPH-induced oxidative hemolysis (Table 3). Among plant extracts, parsley exhibited the highest protective effect (HT_50_ = 273.64 min) and celery recorded the lowest effect (HT_50_ = 194.71 min). All extracts displayed high HT_50_ values compared to the AAPH incubated alone with blood (HT_50_ = 71.34 min). Moreover, plants extracts showed a tremendous protective effect more than the trolox (HT_50_ = 157.54 min) being the reference antioxidant.

Concerning the stabilizing effect (Table 4), the overall analyzed extracts exhibited considerable (*p* < 0.001) stabilizing potential on the erythrocytes membrane. Parsley displayed the highest stabilizing effect (HT_50_ = 301.24 min), while celery recorded the lowest effect (HT_50_ = 228.53 min), which is higher compared to erythrocytes incubated without the extract (control) (HT_50_ = 142.48 min).

### 3.4. Correlation between Different Assays

A correlation analysis (Pearson's correlation square coefficient (*R*^2^), Table 5) was performed to evaluate the link among results of different antioxidant assays. Significant correlations (*p* < 0.001) were depicted (*R*^2^ ≥ 0.935). The lowest correlations were concluded between the AAPH-induced hemolysis test and other tests (*R*^2^ ≤ 0.600).

Outcomes of antioxidant abilities were also correlated to the content of polyphenols measured using Folin–Ciocalteu's method. Results with DPPH, ABTS, and FRAP tests can be linked considerably (*p* < 0.001) to the results found by Folin–Ciocalteu's method (*R*^2^ = 0.935, *R*^2^ = 0.919, and *R*^2^ = 0.910, Table 5). Similarly, a strong relationship was concluded between the iron reducing potential assessed by FRAP and phenol amounts (*R*^2^ = 0.935, Table 5). The weak relationships were observed with TBARS and AAPH tests (*R*^2^ = 0.877 and *R*^2^ = 0.394, respectively).

CT: condensed tannins; MPE: membrane-protecting effect; MSE: membrane-stabilizing effect; Pearson's correlation significance levels are shown with asterisks ^*∗*^*p* < 0.05; ^*∗∗*^*p* < 0.01.

## 4. Discussion

Many reports and randomized controlled trials have shed light on the relationship between polyphenol-rich diets and prevention against various disorders such as diabetes, cancer, and cardiovascular and inflammation-related conditions [27]. The antiradical activities of phenolic compounds, including flavonols, flavonoids, coumarins, and tannins among others, are well documented [28]. In the current study, we have addressed phenolic, flavonoidic and tannin levels plus antioxidant activities of three *Apiaceae* species known as coriander, parsley, and celery, harvested from the Tinghir region in the southeast of Morocco. The level of such compounds contained in plants is usually different. Parsley exhibited high amounts of polyphenol, followed by celery and, then, coriander. Tang et al. [29] and Jung [30] reported high amounts of phenolic compounds in celery (4.640 g GAE.100 g^−1^) and parsley (4.231 g GAE.100 g^−1^), respectively. Sriti et al. [31] measured different polyphenol contents in coriander seeds (1.555 g GAE.100 g^−1^), whole fruit (1.210 g GAE.100 g^−1^), and pericarp (0.292 g GAE.100 g^−1^). Regarding other classes of polyphenols, Msaada et al. [32] have found lower flavonoid content in Syrian coriander (0.251 g CE.100 g^−1^), Egyptian coriander (0.207 g CE.100 g^−1^), and Tunisian coriander (0.203 g CE.100 g^−1^). In addition, the same author reported lower condensed tannin content in Egyptian coriander (0.016 g CE/100 g DW), Tunisian coriander (0.009 g CE/100 g DW), and Syrian coriander (0.017 g CE/100 g DW). These variations could be related to the phytochemical profile, plant part, genetic diversity, geographical distribution, conditions of cultivation, amount of fertilizers, and extraction procedure [16, 33]. Flavonoids are the prevailing class of polyphenols, and they have been mentioned as Nutraceuticals and found to possess a huge number of benefits including antioxidant, antimicrobial, antiulcer, antineoplastic, hepatoprotective, and cardioprotective effects [34]. The HPLC analysis revealed that the three *Apiaceae* species have different phenolic and flavonoid profiles. p-Coumaric, chlorogenic, caffeic acids, luteolin, and quercetin were the prevailing compounds retained by these extracts. Our findings are in agreement with those reported by Yao et al. [35] and Chaowuttikul et al. [36] who have analyzed eleven Chinese and two Thai celery cultivars and found caffeic, ferulic, and p-coumaric acids, as well as luteolin and chlorogenic acid, respectively. However, Hostetler et al. [37] did not detect quercetin and rutin in the parsley extract while luteolin was found at the same amount. Abu-Serie et al. [38] showed that parsley retained all the tested phenolic acids except syringic acid. Moreover, Bilge Ertekin et al. [39] have confirmed that parsley contained caffeic, ferulic, chlorogenic, gallic, and p-coumaric acids. On the other hand, Msaada et al. [32] have demonstrated that chlorogenic, ferulic, p-coumaric, caffeic, vanilic acids, quercetin, rutin, and luteolin are the prevailing compounds of Tunisian and Syrian celery cultivars, whereas the Egyptian cultivar was shown to retain apiginin, quercetin-3-rhamnoside, rosmarinic acid, gallic acid, and trans-hydroxycinnamic acid as the major compounds. Although the current work exhibited similar phenolic profile, the concentration of each compound was different compared to those reported previously. These discrepancies between plants extracts in terms of aerial parts phenolic composition might be ascribed to numerous factors such as genetic variations, cultivation conditions, degree of ripeness, climatic conditions, water availability, plant diseases, harvesting periods, geographic source, storage state, fertilization, soil category, and climatic conditions, as well as the extraction system and analytical method [16].

Phenolic compounds contained in plant extracts have variant antioxidant effects [28]. The quantities of total polyphenols measured by HPLC were considerably reduced than those found by the Folin–Ciocalteu technique. This result might be ascribed to the poor discrimination of the Folin–Ciocalteu reagent, as it reacts positively with different antioxidant compounds (phenolic and nonphenolic substances) [40]. Besides, we have analyzed ten polyphenolic compounds though there is a huge number of polyphenols.

To date, there is no relevant method to evaluate the antioxidant activity; thus, we used DPPH and ABTS radicals-scavenging ability assays, as well as iron-reducing capacity (FRAP). Their principle is based on the ability of an antioxidant to reduce a free radical leading to a change in the intensity of color after being reduced [41]. The intensity of color is oppositely related to the antioxidants level [41]. We concluded that parsley, coriander, and celery extracts possessed both scavenging and reducing powers, but not at the same level. Parsley showed a marked scavenging ability and reducing power among other plants. Given the diversity of mechanisms by which antioxidants act, employing one type of antioxidant capacity evaluation is considered insufficient. As a result, TBARS assay was performed to investigate the antiperoxidative effect on lipids. In fact, during radical attack, free radicals alter lipid membranes, leading to the release of the MDA end product [42]. The latter binds to a chromogenic reagent TBA forming MDA-TBA complex, the latter is quantified spectrophotometrically [42]. Unlike the TBARS assay, the AAPH-induced hemolysis test gives an idea about the antihemolytic potential. Erythrocytes are the first target to radical attack because of two potential sources of ROS depicted by their membrane rich of multiunsaturated fat acids and the hemoglobin redox reactions linked to O_2_ transfer [43]. Due to thermal decomposition, the azo compound AAPH produces free radicals, which attack red blood cells and activate the oxidative chain reactions of macromolecules such as lipids, altering the membrane integrity and, consequently, cause hemolysis [44]. Antioxidants from plants quench free radicals and, thus, protect lipid membranes from radical attack. The protective effect is measured by the vhalftime of hemolysis (HT_50_ which corresponds to the necessary time to the hemolysis of half initial erythrocytes). The more antioxidant levels in the plant extract, the more prolonged the HT_50_ value. In our study, the parsley extract exhibited the strongest antihemolytic activity supported by high erythrocyte membrane-stabilizing and -protecting effects.

The analysis of correlation evidenced significantly strong interrelations (*p* < 0.01) between phenolic content and antioxidant assays (*R*^2^ ≥ 0.910). These interrelations indicate that phenols are the main providers of the observed antioxidant activity and protective effect against lipid peroxidation. As a matter of fact, given the significant antiperoxidative power within the analyzed extracts proved by TBARS assay, which is the first step prior to the evaluation of hypolipidemic activity, they deserve a focused study of their antiatherogenic effect against various cardiovascular diseases.

Our results confirm previous studies on the impacts of phenolic on the scavenging potential of various plant extracts [45, 46]. As a matter of fact, the strong correlation found between the flavonoid content and membrane-protecting effect (*R*^2^ = 0.927) suggests that flavonoids are the main providers of the antihemolytic capacity as already mentioned by Huang [47]. Several authors have reported that flavonoids and other polyphenols can bind to membrane phospholipids, thus protecting membranes against fat deterioration [48, 49]. On a different side, plant extracts induced an increase in the erythrocyte membrane resistance and halftime of hemolysis, which can be related to the high correlation between the flavonoids content and membrane-stabilizing effect on erythrocytes treated only with plant extracts (*R*^2^ = 0.948).

Different results were obtained using various tests including DPPH, ABTS, FRAP, TBARS, and AAPH-induced hemolysis assays. Strong interrelations were noticed between DPPH, ABTS, FRAP, and TBARS assays, while weak correlations were found among the antihemolysis test and others. These differences obtained for the AAPH assay could be explained by the thermodynamic, kinetic properties of the test, and the stereoselectivity of antioxidant compounds [50] plus the substrate and reaction medium could possibly be involved.

Based on this study, the analyzed *Apiaceae* species, mainly parsley, may be a beneficial supplement for use to treat various oxidative burst-mediated disorders via their antiradical properties comprising scavenging ROS, preventing lipid peroxidation and protecting plus stabilizing erythrocyte membranes.

## 5. Conclusions

Parsley, celery, and coriander are very important aromatic and medicinal plants with multiple uses in folk healing. Parsley had the richest phenol and flavonoids contents and, thus, showed potent antioxidant and antiperoxidative activities, followed by celery and, then, coriander. Regardless of their therapeutic effect, they are important sources of polyphenols and effective against free radicals detrimental to the human body. Parsley was able to prevent erythrocytes hemolysis and, thus, showed significant antihemolytic activity followed by coriander and, then, celery. These results could be linked to significant quantity of phenolic compounds such as luteolin, quercetin, p-coumaric, chlorogenic, and caffeic acids present in these plants extracts, especially parsley, which showed the most important antioxidant, antiperoxidative, and antihemolytic properties among the examined plant species. These findings could justify the traditional use of these *Apiaceae* species as medicinal means against many ailments in the Moroccan pharmacopoeia.

## Figures and Tables

**Table 1:** Phenolic and flavonoid profile determined by HPLC in the three *Apiaceae* species extracts (mg/100 g DW).

	Celery	Coriander	Parsley
*Phenolic acids*			
Caffeic acid	67.65 ± 7.04^a^	41.09 ± 5.14^b^	85.67 ± 6.03^c^
Chlorogenic acid	102.33 ± 10.02^d^	82.30 ± 7.51^c^	171.30 ± 10.55^b^
p-Coumaric acid	114.30 ± 10.87^b^	91.18 ± 3.39^a^	96.33 ± 8.08^d^
Ferulic acid	21.67 ± 3.56^c^	16.37 ± 1.45^d^	27.07 ± 7.36^a^
Gallic acid	24.36 ± 3.09^a^	29.16 ± 3.23^b^	37.49 ± 7.50^c^
Syringic acid	6.62 ± 0.93^d^	13.79 ± 1.17^a^	9.49 ± 0.77^c^
Vanillic acid	4.50 ± 0.99^b^	22.11 ± 2.59^c^	26.31 ± 4.16^d^

*Flavonoids*			
Luteolin	31.63 ± 3.51^c^	27.98 ± 2^d^	24.17 ± 5.94^b^
Quercetin	25.08 ± 3.12^a^	6.66 ± 0.30^c^	19.89 ± 3.27^d^
Rutin	16.73 ± 2.05^c^	19.67 ± 2.89^b^	22.03 ± 3.55^a^

Values are averages of triplicate measurements (*n* = 3) ± standard deviation.

**Table 2:** Antioxidant activity of studied plant species based on total phenolic, ﬂavonoids, and condensed tannin levels (g GAE/100 g DW).

Plant species	Total phenolic content *g* GAE/100 g DW	Total flavonoid content *g* RE/100 g DW	Condensed tannins content *g* CE/100 g DW
Coriander (*C. sativum*)	1.372 ± 0.096^c^	1.024 ± 0.081^b^	0.123 ± 0.027^c^
Parsley (*P. crispum*)	2.163 ± 0.104^a^	1.573 ± 0.083^a^	0.308 ± 0.034^b^
Celery (*A*. *graveolens*)	1.739 ± 0.089^b^	0.814 ± 0.061^c^	0.365 ± 0.038^a^

Amounts are mean (*n* = 6) ± SE. Numbers in the identical column followed by various lower-case letters (a–c) are significantly different (*p* < 0.001).

**Table 3:** Antihemolytic activity and membrane-protecting effect induced by plant extracts.

	Half-time of hemolysis (min)	Membrane-protecting effect (%)
Control	142.48 ± 4.14^e^	
AAPH + blood	71.34 ± 4.06^f^	−49.93
AAPH + blood+ (*A*. *graveolens*) extract	194.71 ± 4.16^c^	+36.65
AAPH + blood+ (*C. sativum*) extract	224.54 ± 4.81^b^	+57.59
AAPH + blood+ (*P. crispum*) extract	273.64 ± 4.60^a^	+92.06
AAPH + trolox 1%	157.54 ± 4.36^d^	+10.57

Amounts are mean (*n* = 6) ± SE. numbers in the identical column followed by various lower-case letters (a–f) which are significantly different (*p* < 0.001).

**Table 4:** Antihemolytic activity and the membrane-stabilizing effect induced by plant extracts.

	Half-time of hemolysis (min)	Membrane-stabilizing effect (%)
Control	142.48 ± 4.14^d^	—
Blood+ (*A*. *graveolens*) extract	228.53 ± 5.08^c^	60.39
Blood+ (*C. sativum*) extract	247.02 ± 4.88^b^	69.16
Blood+ (*P. crispum*) extract	301.24 ± 5.51^a^	111.42

Amounts are mean (*n* = 6) ± SE. numbers in the identical column followed by various lower-case letters (a-d) which are significantly different (*p* < 0.001).

**Table 5:** Correlation between polyphenols, flavonoids, condensed tannins, antioxidant, antiperoxidative, and antihemolytic activities.

	Polyphenol	Flavonoid	CT	FRAP	ABTS	DPPH	TBARS	MPE	MSE
Polyphenol	1								
Flavonoid	0.491^*∗∗*^	1							
CT	0.412^*∗∗*^	0.000	1						
FRAP	0.935^*∗∗*^	0.509^*∗∗*^	0.446^*∗∗*^	1					
ABTS	0.910^*∗∗*^	0.579^*∗∗*^	0.390^*∗∗*^	0.988^*∗∗*^	1				
DPPH	0.919^*∗∗*^	0.396^*∗∗*^	0.546^*∗∗*^	0.980^*∗∗*^	0.956^*∗∗*^	1			
TBARS	0.877^*∗∗*^	0.675^*∗∗*^	0.263^*∗*^	0.935^*∗∗*^	0.960^*∗∗*^	0.891^*∗∗*^	1		
MPE	0.394^*∗∗*^	0.927^*∗∗*^	0.007	0.419^*∗∗*^	0.485^*∗∗*^	0.313^*∗*^	0.600^*∗∗*^	1	
MSE	0.504^*∗∗*^	0.948^*∗∗*^	0.002	0.541^*∗∗*^	0.613^*∗∗*^	0.431^*∗∗*^	0.724^*∗∗*^	0.948^*∗∗*^	1

## Data Availability

No data were used to support this study.
